# Development and Validation of an *In Vitro* Resazurin-Based Susceptibility Assay against *Madurella mycetomatis*

**DOI:** 10.1128/AAC.01338-20

**Published:** 2021-02-17

**Authors:** S. O. Abd Algaffar, A. Verbon, W. W. J. van de Sande, S. A. Khalid

**Affiliations:** a Faculty of Pharmacy, University of Science & Technology, Omdurman, Sudan; b Department of Medical Microbiology and Infectious Diseases, Erasmus MC, Rotterdam, The Netherlands

**Keywords:** MIC, amphotericin B, *in vitro* susceptibility, itraconazole, *Madurella mycetomatis*, mycetoma, resazurin, terbinafine, XTT

## Abstract

We present an *in vitro* susceptibility assay for Madurella mycetomatis hyphae using resazurin for endpoint reading. Using this assay, reproducible MICs were obtained for amphotericin B, itraconazole, voriconazole, posaconazole, terbinafine, and micafungin. Results were comparable with those of a 2,3-bis-(2-methoxy-4-nitro-5-sulfophenyl)-2*H*-tetrazolium-5-carboxanilide salt (XTT)-based susceptibility assay. The lowest MICs were obtained for itraconazole and posaconazole (MIC_50_, 0.016 µg/ml) followed by voriconazole (MIC_50_, 0.063 µg/ml). Amphotericin B, micafungin, and terbinafine appeared much less effective.

## Abstract (precis)

We present an *in vitro* susceptibility assay for Madurella mycetomatis hyphae using resazurin for endpoint reading. Using this assay, reproducible MICs were obtained for amphotericin B, itraconazole, voriconazole, posaconazole, terbinafine, and micafungin.

## INTRODUCTION

Madurella mycetomatis is the most common causative agent of eumycetoma. Unfortunately, none of the antifungals currently available for use seem to be therapeutically effective in eumycetoma caused by M. mycetomatis infection. Antifungal therapy always needs to be combined with drastic surgical procedures (1). Therefore, a need to identify novel antifungal agents against M. mycetomatis infection and *in vitro* susceptibility assays are needed.

Previously, an *in vitro* susceptibility assay for M. mycetomatis isolates was published. In that assay, 2,3-bis-(2-methoxy-4-nitro-5-sulfophenyl)-2*H*-tetrazolium-5-carboxanilide salt (XTT) was used as a viability dye to quantify growth after 7 days of incubation (2). However, the readily soluble, cell permeable, and nontoxic redox indicator resazurin is a viability dye that offers extra advantages in terms of its fast and exact visual endpoint determination (3, 4). Resazurin is nontoxic to cells, even after prolonged incubation with slow-growing fungi like mycetoma. The half-life of the reduced resazurin (resorufin) is ∼10 days (4). Thus, resazurin can be added at the start of the *in vitro* susceptibility assay. Visual endpoint reading using resazurin was possible from the 4th day of incubation. Furthermore, resazurin costs less than XTT, which is a paramount advantage in countries of endemicity.

To validate the performance of the newly developed resazurin-based microdilution assay, the susceptibility of 10 M. mycetomatis isolates from different geographic origins toward six structurally diverse antifungal compounds representing the polyenes (amphotericin B, Bristol-Myers Squibb, Woerden, The Netherlands), triazoles (itraconazole, Janssen Pharmaceutical Products, Beerse, Belgium; posaconazole, Schering-low, Kenilworth, NJ; and voriconazole, Pfizer BV, Capelle aan de Ijsel, The Netherlands), echinocandins (micafungin, Astellas Pharma, Leiderdorp, The Netherlands), and allylamines (terbinafine, Novartis Pharma, Basel, Switzerland) was determined. Resazurin-based results were compared with those obtained with the XTT assay published previously (2). Assays were performed in triplicate. In both *in vitro* susceptibility assays, drugs were dissolved in sterile dimethyl sulfoxide (Merck, Darmstadt, Germany). Concentration ranges were as follows: 0.125 to 8 µg/ml for amphotericin B; 0.016 to 1 µg/ml for itraconazole, posaconazole, and voriconazole; 2 to 128 µg/ml for micafungin; and 0.5 to 32 µg/ml for terbinafine. An M. mycetomatis inoculum was prepared in RPMI 1640 medium containing 0.35 g/liter l-glutamine and 1.98 mM 4-morpholinepropane sulfonic acid (5) by sonicating M. mycetomatis mycelia for 10 seconds at 28 µm maximum power (Sonirep 150, Beune de Ronde). After 7 days incubation at 37°C, the mycelia were centrifuged for 5 min at 2,628 × *g* (Rotana 460R, Hettich Zentrifuger, Germany). The medium was poured off, and fresh medium was added. The mycelia were again sonicated for 10 seconds at 28 µm maximum power and adjusted to transmission of 35% to 45% at 660 nm (WPA S800+, Biochrom, UK). A 100-µl standardized hyphal suspension was added to each well of a round-bottom 96-well plate (Greiner Bio-one, The Netherlands) along with 1 µl of the antifungal agent and 1 µl of resazurin solution (0.15 mg/ml). A growth control and a negative control were included. After being added, the readily soluble resazurin formed a deep blue solution. The plates were then sealed and incubated at 37°C for 7 days. During growth, resazurin is transformed into pink fluorescent resorufin in viable metabolically bioactive fungal cells. The quantity of resorufin produced is proportional to the number of viable cells and can be assessed spectrophotometrically or visually without the use of specialized equipment (3). Therefore, the MICs obtained by resazurin assay were determined visually as the first blue/purple well for each agent from the 4th day of incubation. For spectrophotometric endpoints, on the 7th day of incubation, 100 µl of the supernatant was transferred to flat-bottom 96-well plates (Greiner Bio-one). Absorbance was measured at 600 nm using a microplate reader (Epoch 2, Biotek, USA), and the MIC was defined as the lowest concentration of antifungal agent resulting in >80% reduction of viable fungal mass. Percentages of growth inhibition for resazurin and XTT were calculated using the following equations, respectively: percent growth inhibition = 100 − (OD_600_ NC − OD_600_ test/OD_600_ NC − OD_600_ GC) × 100, and percent growth inhibition = 100 − (OD_450_ test − OD_450_ NC OD_450_ GC − OD_450_ NC) × 100, where OD_600_ and OD_450_ are optical density at 600 and 450 nm, respectively.

**FIG 1:**
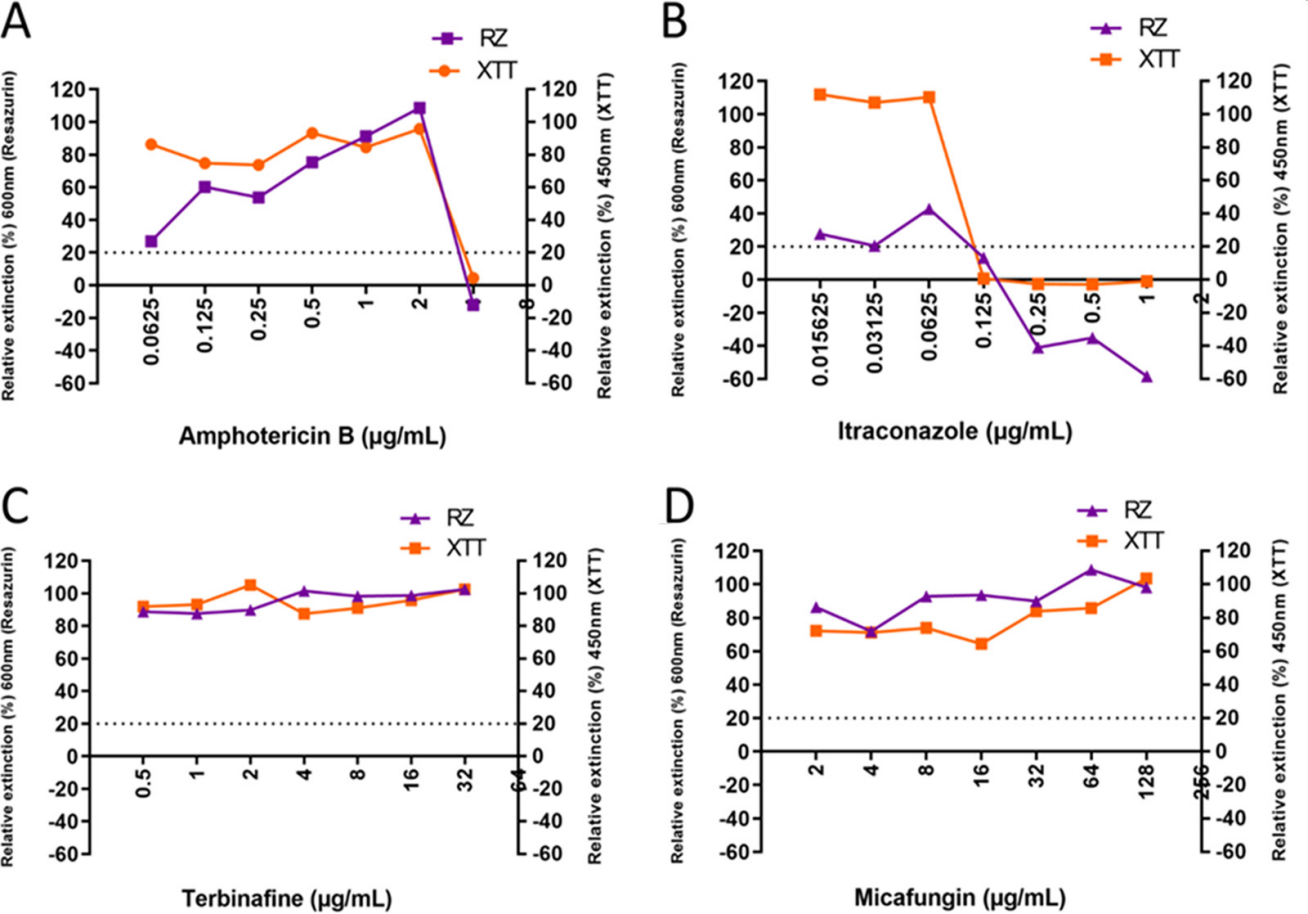
Percent growth of four classes of antifungal agents employing resazurin (RZ) and XTT assays in testing Madurella mycetomatis CBS131320 isolate. (A) Percent growth obtained with XTT and resazurin for amphotericin B. At 4 µg/ml, the percent growth for both resazurin and XTT is below the 20% cutoff line. This is the MIC for this particular isolate. (B) Percent growth for itraconazole. At 0.125 µg/ml, the percent growth for both resazurin and XTT is below the 20% cutoff line. This is the MIC for itraconazole for this particular isolate. (C, D) Percent growth for terbinafine and micafungin. For both drugs, the growth percentages obtained with XTT and resazurin were never below the 20% cutoff line, and no MIC was obtained at the concentrations tested.

As seen in Fig. 1, similar growth patterns were observed for strain CBS131320 with both XTT and resazurin. The first concentration of antifungal agent in which a growth reduction of >80% was observed was considered the MIC (Fig. 1). For strain CBS13120, this was 4 µg/ml for amphotericin B (Fig. 1A), 0.125 µg/ml for itraconazole (Fig. 1B), >32 µg/ml for terbinafine (Fig. 1C), and >128 µg/ml for micafungin (Fig. 1D) using either resazurin or XTT as the viability dye. Employing the newly developed resazurin assay showed a concentration-dependent pattern of antifungal activity for different classes of antifungal agents.

The MICs of the different antifungals used were variable and dependent on the M. mycetomatis isolate. The lowest MIC_50_s were obtained for the azoles, itraconazole, and posaconazole, followed by voriconazole (Table 1). Higher MIC_50_s were obtained for amphotericin, terbinafine, and micafungin. The MICs reported here were comparable with those reported for itraconazole, posaconazole, and voriconazole (2, 5–10). Apparently, inhibition of ergosterol synthesis in M. mycetomatis isolates by either the azole class of antifungal agents or terbinafine offers the best mechanism by which to inhibit M. mycetomatis growth (5, 6). Because there are currently no breakpoints established for M. mycetomatis for any of the antifungal agents tested, the absolute value of the MIC is not indicative of therapeutic success or failure (11–13). As reported in other studies, we found higher MICs for terbinafine (6). However, the performance of terbinafine seemed clinically similar to that of itraconazole. Out of the 68 black-grain eumycetoma cases in Senegal treated with terbinafine and surgery, 20 recovered, 2 had a recurrence, 14 were lost to follow-up, and 32 were still on treatment (12). Based on the 22 patients for whom data were available until the end of treatment, this resulted in a cure rate of 90.9%, which, although lower than the 100% cure rate observed in the same study for itraconazole, was still comparable (12). Amphotericin B appeared to be less effective than azoles at inhibiting M. mycetomatis infection than previously reported (2). It has already been proven that amphotericin B has no promising therapeutic response (14). Apparently, the M. mycetomatis isolates tested were resistant to micafungin, in agreement with earlier studies (7).

**TABLE 1:** MIC distribution for M. mycetomatis isolates and reproducibility and accuracy of the resazurin assay versus the XTT assay

Antifungal agent	M. mycetomatis isolate MIC_50_ (µg/ml [range]) with:		Reproducibility of resazurin system (%)	Agreement (%) for:		
	Resazurin	XTT		Resazurin vs XTT spectrometric	Resazurin visual vs spectrometric	Resazurin visual vs XTT spectrometric
Amphotericin B	4 (1 to >8)	4 (0.5 to >8)	85.2	77.9	88.9	67.7
Itraconazole	0.016 (<0.016 to 0.5)	0.016 (<0.016 to 0.5)	100	88.9	100	77.9
Micafungin	>128 (>128)	>128 (>128)	100	100	100	100
Posaconazole	0.016 (<0.016 to 0.5)	0.016 (<0.016 to 0.5)	96.3	67.7	100	77.9
Terbinafine	>32 (8 to >32)	>32 (8 to >32)	92.3	67.7	77.9	88.9
Voriconazole	0.063 (<0.016 to 0.25)	0.031 (<0.016 to 0.25)	96.3	100	100	100

The assay proved to be reproducible with high percent agreements (Table 1). The percent agreement was determined by calculating the percent readings with ≤1 dilution step difference between the MICs obtained with different methods. The percent agreement between the visual endpoint reading and the spectrophotometric endpoint reading of the resazurin assay ranged between 77.9% and 100%, indicating that visual endpoint reading would be sufficient when spectrophotometric reading of the endpoints is not available (Table 1). To further assess the reproducibility of this assay, a multicenter study should be performed to assess a larger number of genetically diverse isolates.

Our results indicate that resazurin is a good viability dye to be used in *in vitro* susceptibility assays for M. mycetomatis infection. Our *in vitro* susceptibility assay is flexible and can be used to test the antifungal activity of any compound and thus for drug discovery, making this assay more flexible than the commercially available Sensititre YeastOne assay (15), which uses the resazurin derivative alamarBlue for visualization. Unfortunately, the YeastOne assay is only available in premade plates consisting of premade concentration lines for standard antifungals, whereas our assay can be used with any compound of interest.

In conclusion, *in vitro* susceptibility assays for M. mycetomatis based on resazurin as a viability dye offers an extra advantage of visual endpoint reading without resorting to spectrophotometric measurements. The aforementioned advantage coupled with the use of the less costly resazurin, as well as the flexibility with homemade plate layouts, would be an advanced step forward for *in vitro* susceptibility testing against M. mycetomatis compared with MTT, especially in endemic settings.
